# Intervention Mapping of a Gamified Therapy Prescription App for Children With Disabilities: User-Centered Design Approach

**DOI:** 10.2196/34588

**Published:** 2022-08-09

**Authors:** Rowan W Johnson, Becky K White, Daniel F Gucciardi, Noula Gibson, Sian A Williams

**Affiliations:** 1 Therapy Services Ability WA Perth Australia; 2 Curtin School of Allied Health Curtin University Perth Australia; 3 Curtin School of Population Health Curtin University Perth Australia; 4 Reach Health Promotion Innovations Perth Australia; 5 Curtin enAble Institute Faculty of Health Sciences Curtin University Perth Australia; 6 Department of Physiotherapy Perth Children's Hospital Perth Australia; 7 Liggins Institute The University of Auckland Auckland New Zealand

**Keywords:** neurodevelopmental disability, mobile health, self-determination theory, gamification, physiotherapy, occupational therapy, speech pathology, behavior change, mobile phone

## Abstract

**Background:**

Mobile health (mHealth) apps for children are increasing in availability and scope. Therapy (physiotherapy, speech pathology, and occupational therapy) prescription apps to improve home or school program adherence work best when developed to be highly engaging for children and when they incorporate behavior change techniques (BCTs) within their design.

**Objective:**

The aim of this study was to describe the development of a user-centered therapy prescription app for children (aged 6-12 years) with neurodevelopmental disabilities (eg, cerebral palsy, autism spectrum disorder, and intellectual disability) incorporating intervention mapping (IM) and gamified design.

**Methods:**

We used an iterative, user-centered app development model incorporating the first 3 steps of IM. We conducted a needs analysis with user feedback from our previous mHealth app study, a literature review, and a market audit. Change objectives were then specified in alignment with the psychological needs of autonomy, competence, and relatedness identified in self-determination theory. From these objectives, we then selected BCTs, stipulating parameters for effectiveness and how each BCT would be operationalized. A gamification design was planned and implemented focusing on maximizing engagement in children. In total, 2 rounds of consultations with parents, teachers, and therapists and 1 round of prototype app testing with children were conducted to inform app development, with a final iteration developed for further testing.

**Results:**

The IM process resulted in the specification of app elements, self-determination theory–informed BCTs, that were embedded into the app design. The gamification design yielded the selection of a digital pet avatar with a fantasy anime visual theme and multiple layers of incentives earned by completing prescribed therapy activities. Consultation groups with professionals working with children with disabilities (4 therapists and 3 teachers) and parents of children with disabilities (n=3) provided insights into the motivation of children and the pragmatics of implementing app-delivered therapy programs that informed the app development. User testing with children with disabilities (n=4) highlighted their enthusiasm for the app and the need for support in the initial phase of learning the app. App quality testing (Mobile Application Rating Scale-user version) with the children yielded means (out of 5) of 4.5 (SD 0.8) for engagement, 3.3 (SD 1.6) for function, 3.3 (SD 1.7) for aesthetics, and 4.3 (SD 1.1) for subjective quality.

**Conclusions:**

mHealth apps designed for children can be greatly enhanced with a systematic yet flexible development process considering the specific contextual needs of the children with user-centered design, addressing the need for behavior change using the IM process, and maximizing engagement with gamification and strong visual design.

## Introduction

### Background

Mobile health (mHealth) apps designed for children have grown dramatically in availability and scope, coinciding with enhanced accessibility to mobile technologies worldwide (6.38 billion smartphone users in 2021) [1,2]. In 2011, the World Health Organization identified the potential of mHealth technology to transform the delivery of health care and optimize health outcomes [2]. In 2020, the COVID-19 pandemic fast-tracked acceptance of the use of digital technology for augmenting clinical services [3,4], including clinicians who support children with disabilities [5,6]. In this global environment of digital health innovation, mHealth apps are recognized for their potential to augment and enhance clinical care, particularly in terms of their scalability and availability [4].

mHealth apps have the potential to improve adherence to exercise programs in children with neurodevelopmental disabilities (eg, cerebral palsy, autism spectrum disorder, intellectual disability, and Down syndrome) [7]. Children with neurodevelopmental disabilities are often prescribed home and school therapy programs (physiotherapy, occupational therapy, or speech pathology) to increase the amount of therapy activity practice between face-to-face sessions; however, providing programs that children fully engage with at home is challenging for therapists and parents [8,9]. Parents of children with neurodevelopmental disabilities report that children’s adherence to home programs is affected by the clarity of the instructions, the ability to adapt prescribed exercises to individual circumstances, guidance and reassurance with exercise performance, the provision of reminders, collaborative goal setting, monitoring, and the incentivization of adherence [8,10,11]. The potential of mHealth technology to address some of these issues with the use of tools such as exercise videos, adherence tracking, and reminder notifications has been identified [7] but not fully realized. In 1 study, for example, an existing mHealth exercise prescription platform suitable for all age groups but not tailored to children offered little additional benefits compared with traditional paper-based programs for improving exercise adherence in children with disabilities [12]. This finding may be better understood by considering studies that suggest that effective mHealth technology for children demands that app interventions be tailored to their needs and interests and designed to be highly engaging [1,13].

Engagement with digital interventions encompasses both the behaviors of the user (the amount, frequency, duration, and depth of use) and the subjective experiences of the user (ie, attention, interest, and affect) [14]. Apps that are *engaging* are theorized to optimize effectiveness because app use is maintained over time, increasing participants’ exposure to the intervention [13]. Identifying features to increase engagement is an important mHealth app design strategy to enhance long-term behavior change in children [1]. Gamification—the use of gaming elements in a nongaming context [15]—can be a helpful feature for improving engagement with mHealth technology by using intrinsically motivating features such as feedback mechanisms, relatedness support, and autonomy support [16].

Planning behavior change strategies is foundational in the design process for digital health interventions in children [17,18]. Partnerships between app developers and behavioral scientists are needed to use health behavior theory to guide mHealth app development [18]. This partnership enables careful selection of behavior change techniques (BCTs) with consideration of determinants of change as well as the parameters for the effectiveness of each technique [19]. This process is complex as it demands attention to multiple interacting components that often span several spheres of one’s socioecological contexts. Intervention mapping (IM) represents a systematic method of planning behavior change interventions [20] that encourages interventionists to embrace such complexity within the mHealth app development process [21].

### Objective

Incorporating theory-based BCTs and gamification within the design of an mHealth app specifically for children with neurodevelopmental disabilities is indicated to optimize engagement [12]. We reported the app development process for a children’s therapy prescription app designed to optimize engagement and improve program adherence.

## Methods and Results

### App Development Overview

**Figure 1:**
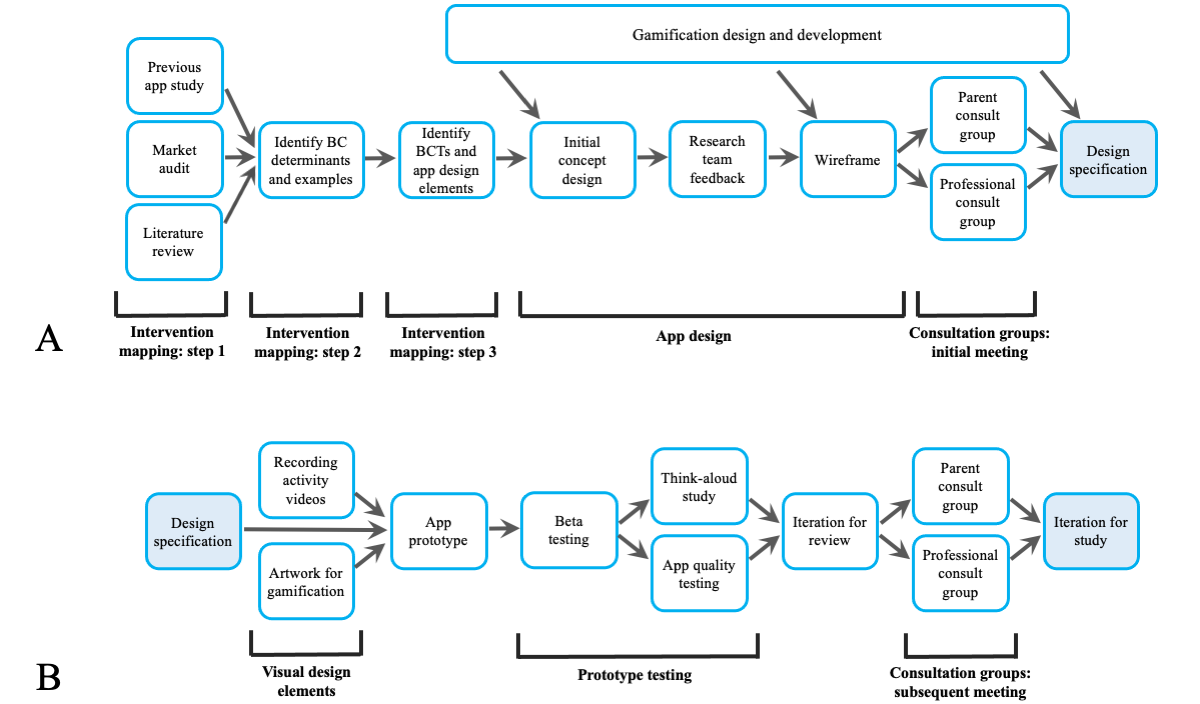
Zingo app development diagram: (A) formative research and (B) design, testing, and iteration. BC: behavior change; BCT: behavior change technique.

An mHealth app was developed from July 2018 to September 2019 based on a 2-stage model by White et al [22] with the goal of enabling children with disabilities to complete prescribed therapy programs. We are a multidisciplinary team with expertise in behavioral science, app development, and exercise and therapy for children with disabilities. The project lead (RWJ) is a physiotherapist with experience in the management of cerebral palsy across the life span and experience in mHealth research for children with disabilities. Our adapted model for the development of the app included several phases (Figure 1), including IM [19,20] to embed BCTs into the app design, development of a video library of children performing commonly prescribed therapy activities, consultation with parent and professional (teacher and therapist) groups at 2 time points, and quantitative and qualitative user testing with children with disabilities.

We used an alternative structure to this paper similar to that of previous manuscripts incorporating IM [23,24], where the methods and results are integrated to maintain a logical and chronological reporting exposition.

### Ethics Approval

Ethics approval was obtained from the Human Research Ethics Committee, Curtin University, Perth, Western Australia (reference HRE2018-0696) before recruitment of the study participants.

### IM Overview

IM is a systematic approach to planning health interventions that guides researchers through a decision-making process incorporating theory, empirical findings from the literature, and analysis of the population needs [20]. IM is supported by a taxonomy of behavior change methods that capture the active ingredients of an intervention, mechanisms of action that link these techniques with theories, and specification of the essential parameters for the effective use of these BCTs [19]. We used the first 3 steps of the IM process: (1) needs assessment, (2) specification of goals and change objectives, and (3) theory-informed methods and applications. These first 3 steps have previously been identified as the most useful in mHealth app development for yielding a program design incorporating both suitable BCTs and methods for implementation [21]; steps 4 to 6 of IM are of particular relevance to broader health promotion programs rather than *“*the technical how*”* [21] of app development, which we implemented using other methods, as described in the following sections and shown in Figure 1.

### IM Step 1: Needs Assessment

Step 1 of IM involves conducting a needs assessment to understand the problem [20,21], which, in the context of our study, was the clinical experience of poor adherence to prescribed therapy programs [7,8].

#### Previous App Study

The needs assessment leveraged findings from our previous work on a commercially available app and web-based platform for exercise prescription (Physitrack). Via a randomized controlled trial involving 46 children with neurodevelopmental disabilities undertaking an 8-week home exercise program, we found that Physitrack did not improve program adherence, exercise performance, or goal attainment compared with conventional paper-based methods [12]. Feedback from parents and physiotherapists of participating children highlighted the limitations experienced using Physitrack for children with disabilities:

Kids liked the technical component, but it probably needs more motivational features.

Physiotherapist 1

It needs increased motivation/rewards/games built into the app...Needs more videos of kids demonstrating exercise. Needs to be more fun.

Physiotherapist 2

The app is boring and does not have any built-in reward for completing each task.

Parent 1

#### Market Audit

A unique element of our needs assessment involved a market audit to understand what exercise and therapy apps targeting children were available. We evaluated their strengths and weaknesses relative to our goal of developing an engaging gamified therapy prescription app for individualized home and school programs. The search was not intended to be exhaustive but to provide an understanding of the market. In July 2018, we identified iOS apps on the App Store targeted for physiotherapy, occupational therapy, and speech pathology for children by entering the following search terms: (“children” OR “kids”) AND (“physiotherapy” OR “physical therapy” OR “speech therapy” OR “occupational therapy”). We also considered apps that the research team had come across in clinical and general experience (eg, GoNoodle). We chose to focus on iOS apps rather than Android apps as this work was funded to develop a tool for school therapy programs for children aged 6 to 12 years. iOS tablets (iPads) are known to be ubiquitous in local primary schools (including kindergarten to grade 6) in our geographical context (Perth, Western Australia), which was later confirmed by teachers in our consultation groups.

A total of 34 apps were identified, of which 22 (65%) were speech pathology apps (targeting articulation; none targeting language development); 10 (29%) targeted physical activity, exercise, and physiotherapy; and 2 (6%) were occupational therapy apps. The identified apps with gamification strategies included physical activity (eg, Biba series and GoNoodle) and articulation (eg, Speech Blubs). The apps identified in this search did not allow therapists to prescribe individualized programs of exercises or therapy activities. In total, 2 children’s apps that did allow some basic exercise prescription (ie, CP-Fit and Sworkit Kids) had limitations relating to exercise selection or ability to customize parameters, and gamification was absent. We did not identify any apps that provided an individualized and customizable therapy prescription for children with disabilities or any apps incorporating recognized BCTs (even those with associated publications), and there were no apps for multidisciplinary therapy programs.

#### Literature Review

A literature review to inform the needs analysis for IM step 1 was conducted to identify key learnings from studies on mHealth apps for children. The review was conducted by 1 author, RWJ, with oversight from DFG using search terms referencing the following 4 elements: apps, mobile technology, children, and therapy (physiotherapy, occupational therapy, or speech pathology). This search, conducted in August 2018, yielded 213 papers. Of 213 papers, screening of titles removed 77 (36.2%) papers (duplicates, lecture notes, viewpoint articles, and commentaries), and the remaining 136 (63.8%) abstracts were reviewed (RWJ) and classified into the following categories: relevant original research, relevant reviews, or nonrelevant. Articles deemed to be relevant met the following criteria: the participants were aged 0 to 17 years; the primary intervention used mobile or web-based technology; and they included promotion of therapy interventions (physiotherapy, occupational therapy, or speech pathology), physical activity, exercise, or behavior change for health promotion. Despite our target audience being children aged 6 to 12 years, publications that included both children and adolescents (ie, a broader pediatric age band) were included to scope the breadth of the available literature; however, where papers were able to draw separate conclusions for adolescents and children [1,18], we were informed by the recommendations for children only. We excluded publications that targeted adult behavior (eg, perinatal care and breastfeeding), other technology (eg, virtual reality, computer software, and communication devices), physical activity and nutrition outcomes without behavior change analysis, specific medical conditions or procedures (eg, health screening, pharmacological studies, and hospital care), studies on general mobile technology use not on mHealth apps, or research not focused on technology or apps (eg, technology just used as an adjunct to the research process). A total of 37 publications were included in the final review, of which 8 (22%) were reviews and 29 (78%) were original research papers. Of the 37 reviewed publications, a summary of 9 (24%) that were deemed the most relevant (to our target group) and informative publications (to behavior change in children delivered via mHealth apps) is provided in Multimedia Appendix 1 [1,13,18,25-30], which illustrates how the literature informed our (IM step 1) needs analysis.

This review highlighted several important considerations for this project. Modeling behavior, promoting practice, and social support appear to be important BCTs that can be incorporated into mHealth for children [18]. However, there is a disconnect between the most frequently used BCTs in mHealth apps and those that are known to be the most effective in children: social support and modeling [1]. The literature highlighted low levels of engagement with mHealth apps among children [1]. Common solutions identified to improve engagement with an app included (1) tailoring the app to specific population groups, (2) having a gamified app design, (3) incorporating elements to personalize the app, and (4) a variety of content and rewards to incentivize the target behavior [1,26-28].

This literature review revealed the significance of embedding key BCTs known to be effective for children while also using engaging gamification elements to drive behavior change toward increasing activity participation in children. These key findings from the literature review were particularly useful for informing the needs analysis and were incorporated into IM step 2, in particular in determining the performance objectives.

### IM Step 2: Specification of the Goal and Change Objectives

In the specification of the goal and change objectives [20,21] conducted by 2 of the authors (RWJ and DFG), 4 performance objectives were selected for the mHealth app focusing on its purpose of engaging children in regular therapy activity practice (Table 1).

Self-determination theory (SDT) was selected to guide the process of identifying behavioral determinants (based on the needs of the population and purpose of the intervention from step 1) and progressing toward selecting BCTs. SDT was selected because of its focus on motivational quality versus quantity, in which the reasons for performing a behavior lie on a continuum from purely intrinsic to extrinsic regulation [31]. We selected the psychological needs as the targets for the following behavior determinants: *autonomy* (“individuals’ propensity to self-organize their behavior and to act in accordance with their integrated self”) [32], *competence* (“the capacity to have an effect on the environment and to attain valued outcomes within it”) [32], and *relatedness* (“the desire to feel connected to others, be loved and cared”) [32]. To complete IM step 2, examples of the desired change in behavior were selected for each of the performance objectives in each of the 3 determinant fields (Table 1), and these informed IM step 3.

**Table 1:** Intervention mapping step 2: matrix of performance objectives and behavior determinants.

Performance objectives	Theory-informed determinants		
	Autonomy	Competence	Relatedness
Child engages in learning about performance of prescribed therapy activities^a^	Child is curious about therapy activities^a^ and how to implement them as recommended Child seeks out information about activity^a^ performance available in the app	Child is confident of attaining required information of therapy activity^a^ performance in the app Child is capable of navigating the app with minimal external supports	Child feels supported to learn via the app from social agents^b^ Child is encouraged to ask questions about desired performance of activities^a^ from social agents^b^
Child physically participates in the therapy program	Child self-initiates app use with therapy activities^a^ (with a level of independence expected for age and ability) Child chooses to participate in the preparation of the environment for activities^a^ (eg, furniture setup, exercise equipment, or activity resources)	Child is guided by prescribing therapists to participate in activities^a^ that are graded to physical capacity Child has appropriate expectations for performance based on knowledge of their physical capacity	Child feels supported by key social agents regardless of the effort enacted or outcomes of participation in the therapy program Child feels valued based on social agents seeking child’s feedback on their experience of activity^a^ performance
Child cognitively participates in the therapy program	Child demonstrates interest in engaging with the app and the therapy program embedded therein Child appreciates the importance of engaging with the therapy program using the app Child demonstrates choice-making in how they engage (or not) in therapy program activities^a^	Child demonstrates ability to engage directly with the app functions (or, where prevented by physical impairment, to communicate to social agent how they want them to engage with the app as a proxy) Child communicates their expectations of extent of following therapy activities^a^ and app engagement Child communicates their personal successes and challenges with completing the therapy program	Child feels valued as the app enables social agents to seek out their contributions (eg, preferences) to the therapy program Child is encouraged to ask questions about therapy program activities^a^ Child has the opportunity to celebrate achievements with peers or key social agents^b^
Child reviews activity^a^ performance and experiential participation	Child self-identifies problems or difficulties faced with completing the program (with guidance on self-reflection offered by social agents^b^) Child communicates what activities^a^ or app elements they enjoyed and why	Child has the opportunity to express success, challenges, and problems to parents, educators, or therapists Child knows how to access summary of activity^a^ completion, feedback on performance, and gamification elements	Child feels supported in understanding challenges Child experiences that their active participation is recognized

^a^Prescribed therapy activity examples: functional strengthening exercises such as abdominal crunches, balance exercises such as walking on a narrow beam, ball skills such as dribbling a football around cones, fine motor activities such as snipping paper with scissors, activities of daily living such as tying shoelaces, receptive language activities such as following specific instructions with colored blocks, expressive language activities such as describing a hidden object to a person guessing, and literacy skills such as constructing words with jumbled sounds or letter cards.

^b^*Social agents* refers to the child’s parent or guardian, teacher, education assistant, or therapist.

### IM Step 3: Theory-Informed Methods and Practical Applications

Drawing on the taxonomy of behavior change methods for IM developed by Kok et al [19], the BCT taxonomy by Michie et al [33], and the classification of motivation and BCTs by Teixeira et al [34], we (RWJ and DFG) selected pertinent motivationally informed BCTs for each determinant and the parameters for effective implementation stated (Table 2). The plan to operationalize each of these techniques was recorded and then, finally, the app design elements to achieve this were specified (Table 2).

This third step concluded a key process of our app development planning using IM to identify theory-based behavior change strategies that were informed by the literature and could be incorporated into our mHealth app. However, another key element in the app design was concurrently underway: gamification design.

**Table 2:** Intervention mapping step 3: behavior change techniques (BCTs), parameters, and app design elements.

Determinant and BCT		Parameters for effectiveness	Operationalization	App design element
**Autonomy**				
	Goal setting (behavior)	Requires commitment to the goal; feedback on results; challenging but achievable goals; appropriate situational support and context	Children and therapists collaboratively set or agree on a goal in terms of percentage of tasked therapy program completed per week. The goal is reviewed and updated	Opportunity for therapist, after consultation with parent and teacher, to input goal (eg, 80% adherence to program) Therapist receives automated email with percentage of adherence update each week with prompt to update adherence goal as required
	Information about health consequences	Requires presentation of information in ways that are appropriately tailored to individuals with sufficient time to do so (eg, text vs infographic)	Information about the health reasons for each activity and opportunity for adult explanation	Therapist required to enter “Purpose” for each custom-made activity (textbox) or has the opportunity to edit precompleted “Purpose” from activity video library instructions, thereby promoting consideration and discussion of health benefits Purpose appears in activity display for child and parent or teacher
	Provide choice	Requires choice from a collaboratively devised set of options. It includes the decision not to participate, to delay, or to change focus, including changing the timing or pace of outcomes	Child can choose the order in which prescribed activities are tackled and change that order on any day the activities are completed	Child can select any activity from therapist-prescribed program with guidance from parent or teacher as appropriate, thereby facilitating choice of order of activity completion (within a day) Interface specifies frequency of each therapy activity in terms of attempts per week (rather than identifying specific days of the week), thereby facilitating the child’s choice of program structure (across each week) with adult guidance where required
**Competence**				
	Demonstration of behavior	Requires instruction and enactment with individual feedback	Providing visual examples of how to perform activities	Videos of therapy activities: children modeling activity performance in video library Therapist can use custom video of the child themselves modeling the activity and incorporate it into the program
	Self-monitoring of behavior	The monitoring must be of the specific behavior (not a health outcome). The data must be interpreted and used. The reward must be reinforcing to the individual	Providing the opportunity for children to self-monitor and evaluate their behavioral experience and performance as well as initiate feedback discussions with therapists	After each activity completion, the child has the opportunity to reflect and provide feedback on their experience Brief or detailed feedback options available for each activity Multiple feedback options available, including activity difficulty, emoticons, written emotions experienced, and blank textbox Feedback for each activity available to the therapist and to the child and family on their interface for reflection as well as automated duration and frequency data
	Instruction on how to perform behavior	Requires communication with language that suits the learner	Pre-existing activities from library include written instructions on how to perform the specific tasks, or customized activities require therapists to document those instructions	App provides the therapist with the opportunity to customize activity purpose description and instructions
	Feedback on behavior	Requires availability of data and monitoring of behavior; data must be interpreted and used	Children-friendly feedback on task completion and performance	“My Stars” tab with overview of weekly therapy activity performance summary (out of total number of activities per week recommended) Simple visual representation of weekly program completion with circular progress chart incorporating completion goal (percentage) set by the therapist and progress toward 100% completion Prompt in gamified rewards section (“Pet shop”) to review progress in “My Stars” to earn incentives
**Relatedness**				
	Social support (emotional)	Requires availability of positive support	Prompt parents to discuss progress with child and encourage them	Weekly automated email to parent on progress with positive wording and prompt to parent to encourage the child with their achievements and progress
	Material and social rewards (behavior)	Requires tailored rewards and that the rewards are seen as a consequence of the behavior	Providing written reinforcement (social) and valued objects (material) for positive behavioral progress	Gamification incentives when achieving goals, involving interaction with digital pet (pet emotional state progression, pet evolution, and pet purchases)

### Gamification Design

**Figure 2:**
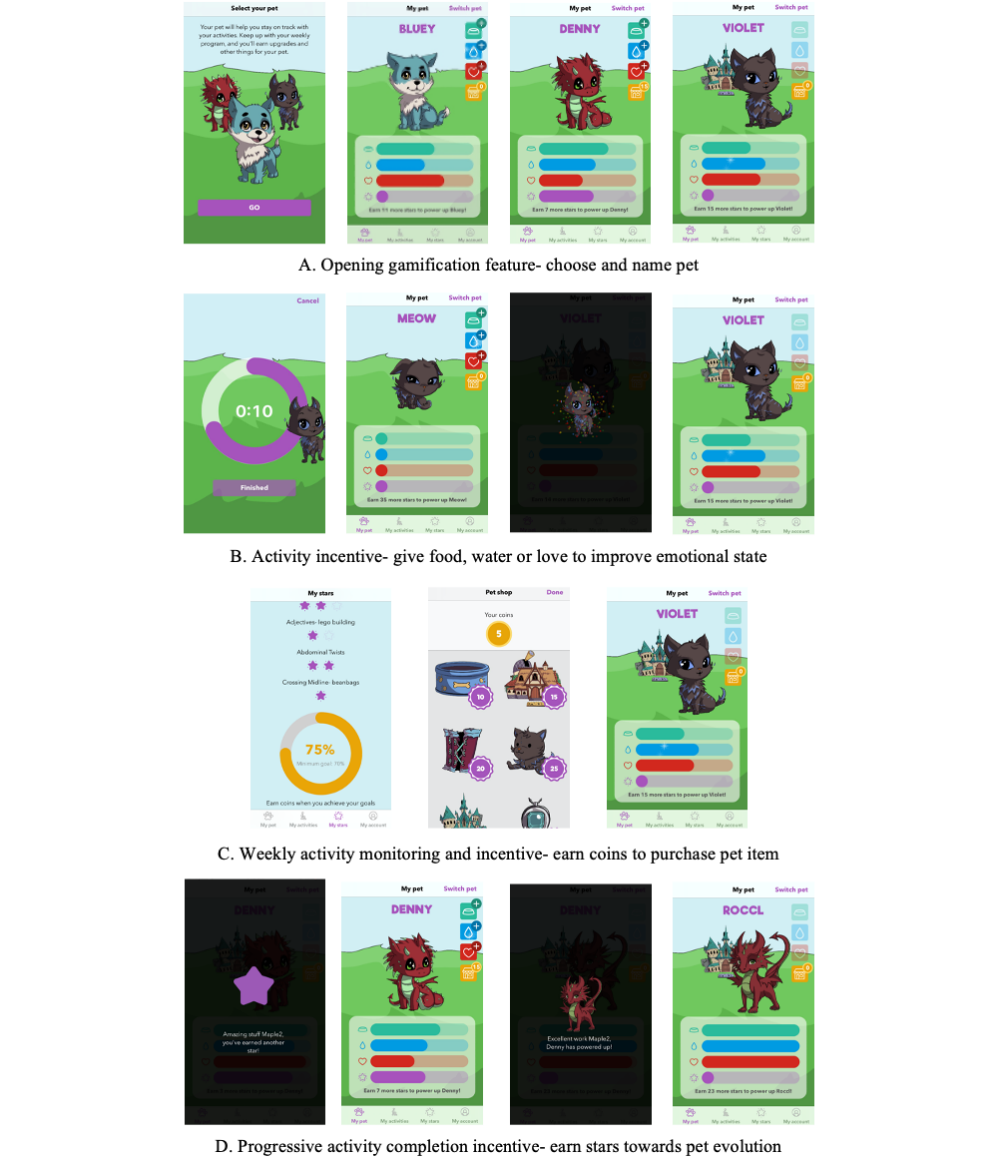
Gamification design features.

The use of mHealth apps that use gamification for promoting health-related behavior change has been investigated across different fields of health [16,35] and is particularly relevant when developing interventions for children [1,16]. Gamified systems have been linked to autonomous motivation, as defined in SDT [36], by using motivational features such as immediate success feedback, continuous progress feedback, and goal setting [16]. The gamification strategy for this app was designed with consideration of the context: supporting children in a classroom or home environment to complete nondigital therapy activities and exercises. A considered decision was made not to develop a full-featured electronic gaming app, known for engrossing children in a state of intense focus (ie, a *flow-state*) where they may have reduced awareness of the world around them [37] and that may be a distraction from therapy activities. Rather, gamification elements that are highly engaging but have a specific start and end were chosen so that the child is able to put down the device and continue with the next therapy activity. We designed several different systems of gamified goals and incentives to keep users engaged [28] (Figure 2). The child using the app can achieve gamified goals and incentives by completing the prescribed therapy activities. Using this strategy, engagement with the app is more likely to be maintained as, once the child achieves 1 gamified goal and incentive, there is a different goal and incentive to work toward.

The gamified systems revolve around looking after and rewarding a digital pet. We chose digital pets as they have previously been used in mHealth gamification design for children [28] and in popular entertainment and games (eg, Pokémon and Tamagotchi). We incorporated a fantasy anime (Japanese animation style) design theme and bright colors into our digital pet design to appeal to children. These concepts around gamification developed progressively, with the basic themes being identified and incorporated into the initial concept design (Figure 1A). Further gamification mechanics were introduced at different points in the development and incorporated into the wireframe and design specification (each described in the following sections) before being actualized in the initial app prototype and refined concurrently with other app elements to the final iteration (Figure 1B).

### App Design

**Figure 3:**
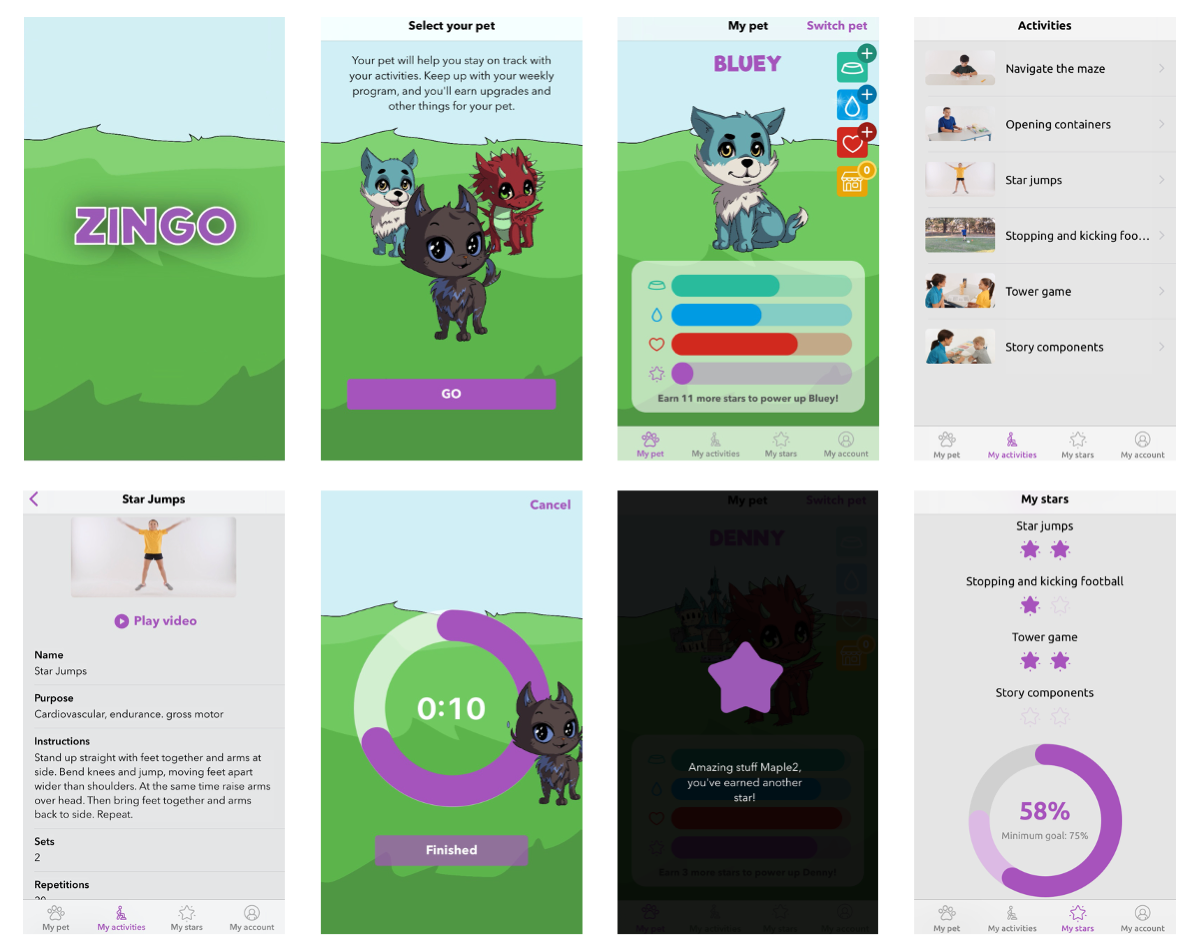
Zingo app user interface overview.

The concept design of the app incorporated sketches of different screens, a description of the functionality of each screen, and a flow chart demonstrating how the screens would be integrated. Concept designs were completed for a therapist interface and a child interface (Figure 3). The therapist interface was for creating and editing individualized therapy programs for multiple children; the child interface is where the BCTs and gamification elements were used. The interface was designed not only to maximize the screen size and functionality of an iPad but also to operate effectively on an iPhone. The research team provided feedback on this initial design to clarify and refine the concept for the next step—the development of a wireframe, which is “a representation of the skeletal structure of a mobile application [outlining the] relationship between the elements that make up a mobile application*”* [38]. The wireframe excludes visual design and graphic elements (to be added later in the app) but provides a semifunctional, basic prototype for early investigation. The wireframe was then brought to our consultation groups for review and feedback.

### Consultation Groups

#### Recruitment

A total of 2 consultation groups were recruited to attend 2 separate meetings each: an education and health professional group and a parent group. We did not recruit children with neurodevelopmental disabilities for our consultation groups; instead, we chose their advocates because of the complexity of the concepts to understand the app and recommend changes, particularly given the real-world context of app use enmeshed with home or school therapy activity performance. We sought the views of children in our testing process (refer *Prototype Testing*) and in the feasibility study that followed the app design.

Potential participants for both groups were identified by word of mouth through existing networks. Once potential participants were identified, they were contacted by a third party (research volunteer) to inform them of the consultation groups and gauge their interest. Interested parties were then contacted by the lead researcher to discuss their role, and those who agreed to participate signed a consent form.

#### Initial Meeting: Wireframe Review

All participants were familiar with using smart devices (phones and tablets) with children in either a parenting or professional context. Consultation group sizes were chosen pragmatically considering the feasibility of recruitment, seeking a diversity of views from a range of professional backgrounds and parental experiences and allowing for in-depth analysis and feedback on the wireframe. For the professional group, 3 teachers working with children with neurodevelopmental disabilities and 4 therapists working for a nongovernment community disability service provider (1/4, 25% speech pathologists; 2/4, 50% occupational therapists; and 1/4, 25% physiotherapists) were recruited. For the parent group, 3 parents of children aged 6 to 14 years with disabilities (1/3, 33% fathers and 2/3, 67% mothers) were recruited through word-of-mouth communication with therapists to identify parents who may be interested. One of the mothers was unable to attend the first group meeting because of illness but attended the second group.

The first meeting was early in the development process to review the app’s wireframe. The second meeting was later in the development process to review an early prototype of the app (Figure 1B). The consultation groups began with the lead investigator taking participants through the relevant app interface and features (on either the wireframe or app prototype), with time for the participants to explore the app and ask questions or provide spontaneous observations and feedback. Subsequently, the participants engaged in a semistructured discussion with set topics covering the purpose and framing of the app for children with disabilities, the app content and BCTs used, gamification and other engagement strategies, and the pragmatics of using the app in a school environment for the purpose of practicing exercises and activities prescribed by therapists for the child. The consultation sessions lasted 60 to 90 minutes.

The initial consultation groups reviewing the wireframe of the app provided important insights into motivation for children, support systems, and gamification recommendations. They were also able to outline pragmatic considerations for the implementation of app-delivered therapy programs in the classroom environment. Motivators of children and app features that would promote such motivators were a key theme. Tools such as an in-app timer during exercises with a 3-2-1 (or *ready-set-go*) lead-in were identified by both groups as exciting and were incorporated into subsequent app prototypes. The parents and professionals were enthusiastic about the game-inspired design. The topic of possible types of digital avatars was raised, and parents settled on digital pets as being motivating:

I think the pet idea would be the most exciting...so the idea of looking after a pet, ’cause [children with disabilities] tend to be more nurturing, the ones that I’ve met.

Parent 1

I’d go with pets as well. So, I’ve got two girls and they play a lot of games involving pets, so “Animal Jam” is one of them...my one daughter she’s 12, who’s got a disability, but she loves this game...I think she likes the characters and she likes the fact that you can change them, they can evolve over time.

Parent 2

The parents also alluded to a digital pet being superior to an animated child avatar because of potential issues with self-perception for children with disabilities—they might not fully identify with an avatar of a child who appears able-bodied (or avatars with visual indicators of disability may not appeal to children with mild disabilities). This issue of self-perception also came up when discussing a potential function for the app to collect a video of the child performing a prescribed activity as a feedback mechanism:

I know my daughter doesn’t like looking at herself.

Parent 2

This feedback aligns with previous work investigating physical activity participation in people with disabilities, which identified poor self-perception as a common barrier to participation [39] but also feelings of improved self-worth when they do participate [40]. In terms of the pragmatics of using this app in the classroom, when the interviewer raised the topic of the potential for an app to be so engaging that it distracts from active therapy or educational time, the parents acknowledged this potential but conversely spoke of the need for the child to have some time to be fully engaged in the app and “fall in love with it*”* (parent 1) so that it might improve their motivation to practice. Similarly, the teachers identified that some students would lose interest if they could not hold the iPad and spend some time interacting with it themselves.

Adult social support for children practicing therapeutic exercises was discussed, and the parents suggested that the app could provide an update for parents on their child’s progress in performing activities at school. This could best be delivered as a weekly email with a simple graphic summary. Teachers who provide active support to the child in the classroom were clear that they did not want an *alert* or *help* feature that would generate noise as the teacher and aides would always be attentive to the child’s needs. The teachers and therapists were also able to specify some key features that they thought were important (eg, a feature to generate a printable PDF of the program) or unhelpful (eg, incorporating reminder alerts or notifications when flexible use of the app was described as preferable in the classroom).

### Design Specification for Prototype App

Feedback, concepts, and functionality requirements derived from the parent and professional consultation groups who reviewed the wireframe were amalgamated with the app design elements identified from the IM process (steps 1-3). These were considered along with feedback on the wireframe from the research team and developer to formulate the design specifications for the app (Figure 1A) and build the first prototype of the app. Ongoing communication between the developer and research team was maintained to clarify the functional tools, interface layout, and other app function elements.

### Visual Design Elements

**Figure 4:**
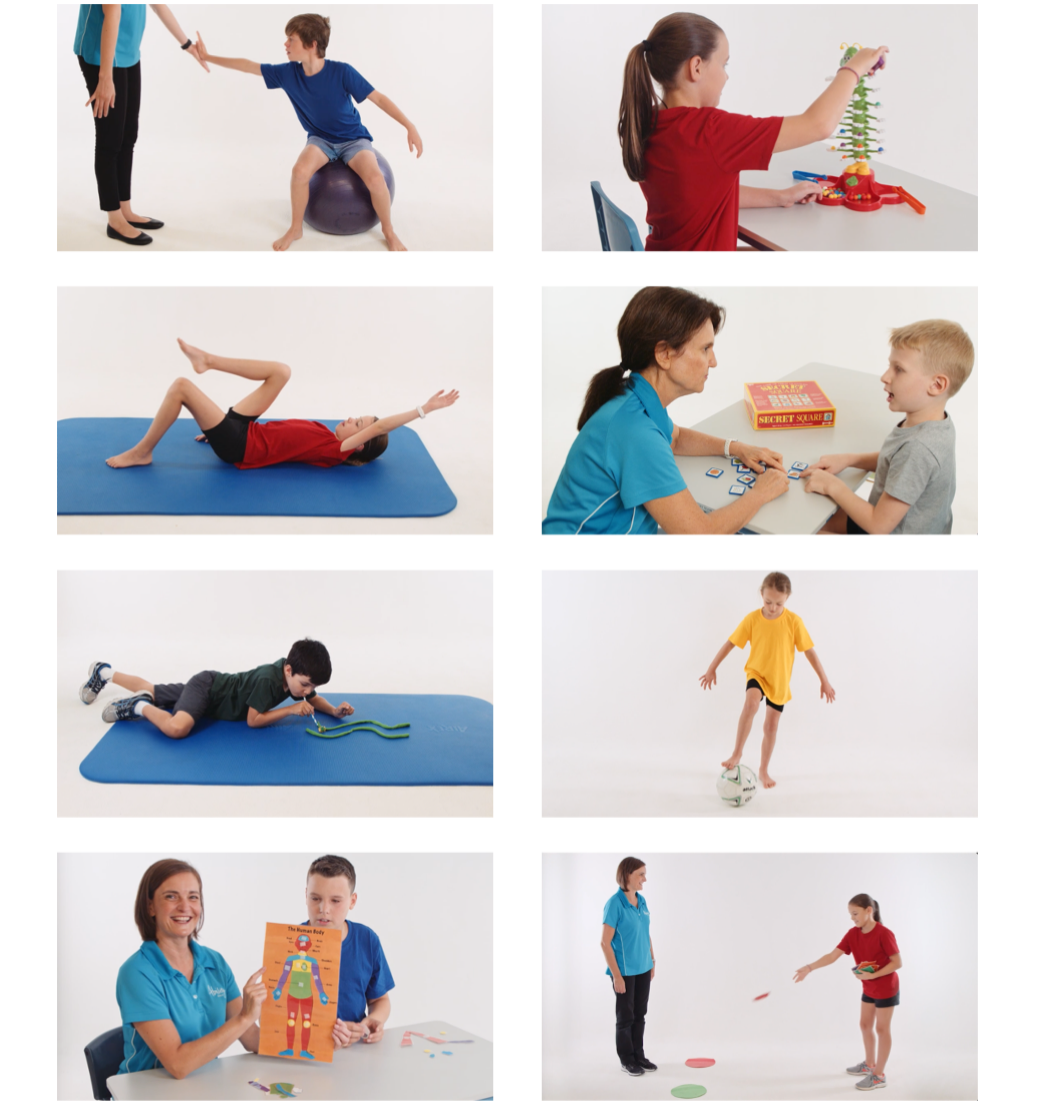
Therapy activity videos: sample of still images taken from videos.

Following the design specifications, we began work on the visual elements of the app to create an engaging app for children. In total, 2 BCTs identified from IM, “demonstration of behavior” and “instruction on how to perform behavior,” along with reflections from participants in our previous study requesting videos with children (rather than adults) [12] informed the development of a library of therapy activity demonstration videos using children as models. A team of experienced therapists (a speech pathologist, occupational therapist, and physiotherapist), including RWJ, worked with a professional videographer and child volunteers to film commonly prescribed therapy activity and exercise demonstration videos (Figure 4) along with written activity goals and instructions. In addition, RWJ worked with a professional artist to develop key images for the gamification elements: background art, drawings of purchasable items (eg, custom pet bowls and fantasy pet houses), and 3 digital pets drawn in anime style. Each pet was drawn with 4 emotional states (sad, content, happy, and elated) and 3 progression states (child-like, maturing, and strong), resulting in 36 pet images for use with game-inspired app design (Figure 2).

### Prototype Testing

All design elements were incorporated into the app prototype (Figures 2 and 3) before user testing, for which there were 2 phases. First, 4 experienced app testers performed beta testing to look for software bugs, crashes, layout issues, and other technical errors. The second testing phase involved user evaluation of the quality, function, engagement, and usability of the app. Recruitment for prototype testing targeted children who met our inclusion criteria of being aged 6 to 12 years; having a neurodevelopmental disability; being interested in testing a new therapy app; having the fine motor skills to use an app and visual acuity to see the screen, including the various app interface elements; and being able to effectively communicate their experiences and opinions. We set the recruitment target at 4 to 6 children as this number of participants is recognized as sufficient to yield 75% of usability problems, with diminishing returns with more participants [41]. Interested and consenting participants undertook a testing protocol over a single session in a private room with a parent present using 2 methods (Textbox 1; Figure 1B).

**Methods for the app testing protocol.**

**Testing protocol methods**

- Think-aloud walk-throughs are an industry-recognized method of testing mobile health apps [42]. RWJ demonstrated the think-aloud process to the children using another app as an example. The participants then opened the app prototype for the first time and were asked to describe what they were doing and their thoughts about using the app. The children were prompted to use the essential features of the app (determined before the walk-throughs) only if they did not access them spontaneously. Several therapy activities were included in the example program for the children to try, and necessary equipment was provided to simulate a real experience of using the app for therapy practice. We voice-recorded and transcribed their *think-alouds*. Transcripts were analyzed by a single reviewer (RWJ) to identify think-aloud content about the participants’ app experiences, including app usability, app navigation, engagement, and emotional expressions.
- The Mobile Application Rating Scale-user version (uMARS) was selected as a broad app quality questionnaire that has submeasures for usability, engagement, aesthetics, information, and subjective quality. Following the think-aloud walk-through process, the children completed the uMARS with the support of their parents and guidance from RWJ. Guidance included reading the question and possible responses out loud and explaining any terms or concepts using language suitable for the child’s age and level of understanding.

We recruited 5 participants for the user testing process, of whom 4 (80%) completed the process and 1 (20%) did not arrive for the scheduled testing date and was lost to follow-up. Implementing the testing processes with the children who volunteered proved challenging. We intended to engage a sample of children representing the target age range of 6 to 12 years, yet the 4 participants who completed the process had a mean age of 6.8 (SD 1.0 years; female: 2/4, 50%; diagnosis: 3/4, 75% cerebral palsy and 1/4, 25% Joubert syndrome); therefore, the children’s cognitive development was a factor, particularly in the think-aloud walk-through. We observed that the children were limited in their capacity to interact with the app and talk about their experiences simultaneously. The limited literacy of children of this age group impeded their ability to navigate the app independently as the in-app pictorial cues are supported by simple written descriptions, and the interviewer needed to read labels and instructions for the children. The children needed prompting to tell the researcher what they were doing and thinking as they used the app, deviating from the intended think-aloud walk-through process of spontaneous verbalization of the technology experience. In addition, one of the children who arrived did not meet the criterion of having effective verbal communication despite previous parent confirmation that they did. Despite these challenges, we obtained helpful information from the 3 participants with effective verbal communication. Initially, the children showed confusion regarding how to progress with the app, and the importance of adult support became evident. Also observed was the children’s enthusiasm for the app after the initial learning process and their perception of it as a gaming app:

I like this game, I want to download it.

Child 1

The children were enthusiastic about trying the therapy activities that had been selected. The 3 children with verbal communication ability completed the relevant sections of the Mobile Application Rating Scale-user version (uMARS) assessment with means (out of 5 for each) of 4.5 (SD 0.8) for engagement, 3.3 (SD 1.6) for function, 3.3 (SD 1.7) for aesthetics, and 4.3 (SD 1.1) for subjective quality. After completing the prototype testing, we prepared an app iteration for review in the final round of consultation groups.

### Consultation Groups—Subsequent Meeting: Prototype App Review

In the second round of consultations, the app prototype was reviewed with a strong positive reception in both the parent and professional groups:

I think the kids will definitely, they [will] definitely like it.

Professional 3

There was a clear theme in the discussions around the process of learning how to maximize the gamification and reward features, with both groups expressing some initial confusion with some aspects of the different pet rewards. Some of these were due to glitches or reward systems not functioning as planned. An aspect of the gamification, *activity completion rewards that improve pet emotional state,* was revealed to be insufficiently responsive to change, whereas some other issues of app mechanics were identified as part of the normal learning process. Additional topics were app esthetics, which received widespread approval in terms of the general interface, activity videos, and gamification elements:

Very beautiful. Good colors, nice and bright. The animals themselves are exactly what I was hoping they would look like...in line with that kind of Pokémon, you know, cuteness.

Parent 1

Feedback was also provided on the interface—it was described as simple and effective, but they identified the need to increase the size of the icons and text in the tab buttons. The parents identified this element as important to meet the needs of children with visual impairment. Excellent advice was provided on how the app could work in terms of actual therapy practice in the school environment and methods of communication of performance at school with parents. The groups also identified the app as highly motivating for children:

It’s so similar to other games that you would have to purchase stuff...[ie, in app purchases].

Parent 1

And that’s the beauty of it. This game you buy things based on the effort you put in, not money, which is great. So, the child says I want...the pendant, well “you need to go and do some more exercises and get it,” and that’s great motivation.

Parent 3

This final consultation group completed the testing and feedback process. We implemented some immediate changes based on the feedback received in the beta testing, user testing, and consultation groups; for example, we improved the responsiveness of the incentives (Figure 2) that affect pet emotional state to yield rapid changes to reassure the user that their efforts were *helping* their pet, and we increased the font size. In later iterations, more substantial changes could be implemented, including an onboarding in-app tutorial to assist users in learning the functions, particularly the systems of pet rewards, and a printable PDF of the therapy program so this could be on display as an additional reminder and aid to do the therapy program. With these changes, the *Zingo* app was completed for further planned evaluation: a feasibility study of a 4-week therapy program using *Zingo* to take place with students aged 6 to 12 years with childhood disabilities in Western Australia.

## Discussion

### Principal Findings

We have reported on the development of an app for the prescription of home and school therapy activity programs for children with disabilities incorporating theory-based behavior change and gamification. We carefully selected BCTs based on SDT using the first 3 steps of the IM process that were then incorporated into the app design. We developed an app that is designed to be engaging and fun for children and effective for therapists based on user consultation throughout the development process and is ready for further evaluation.

### Comparison With Prior Work

Our study is the first to incorporate IM and, more broadly, behavioral science principles into the design and development of a therapy or exercise prescription app for children. We used the first 3 steps of the IM process in a similar approach to Direito et al [21], who used IM steps 1 to 3 to develop an app to increase physical activity in adults, and DeSmet et al [24], who used IM steps 1 to 3 in serious game design to address cyberbullying among adolescents. An app-based parenting program to prevent childhood obesity by Karssen et al [43] used all 6 steps of IM in its design and implementation. The execution of IM steps 1 to 3 in the latter study has foreseeable congruity with our process; however, further similarities can be identified between the implementation of IM step 4 by Karssen et al [43] and the iterative, user-centered development process we used (Figure 1), including that both studies implemented consultation groups with parents and health care professionals to inform app design. Our process went a step further by incorporating specific app testing and feedback from our end users, children with disabilities, into the development process. Furthermore, Karssen et al [43], in IM step 6, implemented a thorough program evaluation via 2 randomized controlled trials, whereas we similarly implemented a specific trial to evaluate the Zingo app with a feasibility study that we will report separately. As an approach to app development, we can see how the IM process for selecting and implementing BCTs in the first 3 steps of IM can be used in conjunction with other methods and that these other processes are complementary to the IM protocol.

We planned and embedded gamification elements in the Zingo app design to maximize the engagement of Zingo app end users—children with disabilities. Some traditional methods of gamification such as leaderboards and badges were discarded to focus on elements that would be highly motivating for children. We incorporated some recognized gamification interface design elements—avatar use and fantasy theme [44]—but modified them for increased engagement in children; for example, the avatar element was modified to a digital pet that does not directly represent the user but draws on interest in animals and anime (ie, Japanese-style cartoon) characters. We used some recognized game mechanics, including clear goals, progression, and immediate feedback [44], along with recognized gamification point systems—experience points and redeemable points [45]—but added themed twists to adapt for children; for example, experience points are earned with every activity completion but are in the form of *stars*. Stars earned by children are tracked on the progress page for each activity and in a graphic representation of overall weekly progress and are also used on the primary gamification page with a star bar that fills up and, when full, the digital pet evolves to a more advanced state. Some of these elements that we used can be defined as “deep gamification,” in which core processes of the app are changed for gamification and a “game design” approach is used to redesign the app to facilitate game mechanics [45]. By contrast, shallow gamification is where the activity is unchanged but enhanced with gamified elements (eg, points and badges) [45]. This approach, along with a targeted design interface for children and adaptation of game mechanics to maximize engagement, can be recommended from our study for future mHealth app design for children.

In the field of physiotherapy, there are feasible gamified apps for children focused on increasing physical activity [29,46,47], but there is a dearth of literature that describes or evaluates gamified apps for prescribing individualized exercise programs for children. In the field of occupational therapy, a high uptake in app use has been reported along with a great diversity of apps and their uses, including fine motor skills, activities of daily living, writing, visual motor skills, and play [48,49]. Some of the referenced apps use engaging and fun elements for children; however, gamification is not directly reported on, and these apps are most frequently used as a direct (ie, face-to-face) intervention tool rather than being designed as a home program tool [49]. For speech pathology, an abundance of apps for children have been recognized, many with gamification elements; however, in existing apps, there is a predominant focus on articulation and phenology (eg, Articulation Station, Articulate it!, and Articulation Scenes) [50], not language development. For example, in a review of 132 speech pathology apps, those for children with language disorders were excluded as they were found to be solely clinical assessment tools; there were no intervention apps targeting language for children to use [50]. These findings support the clinical utility of an app that is specifically for developing home and school programs where each child can be prescribed a unique program by their therapist rather than predetermined activities and that is multidisciplinary in its design, includes language development activities, and uses deep gamification features for an engaging experience for children.

### Strengths and Limitations

Key strengths of this mHealth app design include a comprehensive approach to development and testing, incorporation of app design elements to support the functional use of the app in schools and homes, real-time feedback mechanisms and other features identified by therapists, and app design to address key psychological needs for building intrinsic motivation. Despite the rigor taken in this app development, we identified some limitations in the process that should be considered. Think-aloud testing processes were ineffective among some of the testing users despite our efforts to check eligibility during recruitment; their young age and neurodevelopmental disability meant that they had limited capacity to perform the think-aloud process of describing their thoughts and experiences while using the app prototype. In addition, our other user testing evaluation tool, the uMARS questionnaire, yielded some helpful information, but it required modification and support from the researcher and a lot of prompting from parents in some cases. An app quality rating scale developed specifically for children with disabilities is needed to improve testing.

Zingo is suitable for children with a broad range of neurodevelopmental disabilities, but some children with more severe disabilities will require additional assistance when using the app. When considering suitability, it is important to acknowledge that, in common clinical practice for therapy programs, a child will perform the program with parent, teacher, or caregiver supervision or assistance. Zingo app therapy programs are not intended to be performed completely independently. Another consideration is that Zingo cannot be used without the involvement of a therapist to set up the program; therefore, a therapist will use professional knowledge and parental consultation to determine suitability. To engage physically with Zingo, a child requires sufficient fine motor skills to tap a single *button* (ie, on-screen image) in the app, so the motor demand is not high. In our clinical experience, most children with disabilities can achieve this, with aides if required (eg, postural support seating), particularly when using a tablet device rather than a smaller screen as is common practice for this age group. However, children with severe upper limb contracture and deformity or significant visual impairment may not be able to activate the screen themselves. In these scenarios, the parent or caregiver could complete the fine motor component of pressing *buttons* on the app as a proxy. To cognitively engage with the app, most children with disabilities will need some support from a therapist or caregiver in the initial learning period and varying levels of support after this. To be independent with the app (with only adult supervision), the child requires a moderate level of reading capacity to select the correct options, although, wherever possible, we supplemented words with icons. Children without early literacy will require more parental support throughout the process. The app may be of limited benefit for children with profound intellectual and physical disabilities although, if the parent chooses to use Zingo for the child (eg, showing the screen to the child but pressing *buttons* for them), the child may still find the bright colors, music, digital pet images, and activity videos more engaging than conventional paper-based therapy programs.

### Conclusions

We developed a gamified therapy prescription app embedded with theory-informed BCTs for the delivery and implementation of individually prescribed therapy programs for children with neurodevelopmental disabilities. We can recommend the use of the IM process to select and implement the most effective BCTs. A strong user-centred design process, as outlined here, with testing and feedback at multiple stages was important for adapting the app outcome to best suit the needs of the users and could be effectively used in future mHealth app development projects. We recommend that other methods for user testing with younger children with disabilities be explored in future studies. We prepared the *Zingo* app in this development phase for future study in *real-world* environments; a mixed methods feasibility study is planned to gain a stronger understanding of the *Zingo* app’s function to deliver therapy programs in a way that is fun and engaging for children to improve adherence to those programs and thereby maximize the benefits of regular therapy activity practice for children with disabilities.

## Appendix

Multimedia Appendix 1 Key findings from the literature review on behavior change in children with mobile health interventions for intervention mapping step 1: needs analysis.

## Abbreviations

BCT: behavior change technique
IM: intervention mapping
mHealth: mobile health
NGCS: Non-Government Centre Support
SDT: self-determination theory
uMARS: Mobile Application Rating Scale-user version
