# Supplementary material for: Intervention Mapping of a Gamified Therapy Prescription App for Children With Disabilities: User-Centered Design Approach
Source: JMIR Pediatr Parent. 2022 Aug 9;5(3):e34588. doi: 10.2196/34588 (PMC9399848; doi:10.2196/34588)
Supplement: Multimedia Appendix 1 [file pediatrics_v5i3e34588_app1.pdf]

Multimedia Appendix 1: Key findings from Literature Review on behavior change in children with mHealth interventions, for Intervention Mapping step One: Needs Analysis

Reference	Title	Key findings for IM step One
		Relevant Reviews
Brannon and Cushing [18] (2015)	A systematic review: Is there an app for that? Translational science of pediatric behavior change for physical activity and dietary interventions	• Effective strategies for children to promote physical activity: modelling appropriate behavior (commonly by using exercise videos)<br>• Most important behavior strategies for young children to engage in healthy behaviors: modelling, practice, and social support<br>• Partnerships between technology developers and behavioral scientists recommended
Turner et al [26] (2015)	Prevention and treatment of pediatric obesity using mobile and wireless technologies: a systematic review	• mHealth apps are feasible and acceptable for promoting healthy diet and physical activity; they are perceived as a supportive, motivational and fun compared to traditional approaches<br>• Apps and games that were particularly successful in promoting physical activity when feedback, self-monitoring and social connection was involved<br>• Recommended using a variety of short- and long-term motivational techniques, personalized information, using language that promotes autonomy, using only positive feedback, using an avatar and providing reminders
Schoeppe et al [13] (2016)	Efficacy of interventions that use apps to improve diet, physical activity and sedentary behavior: a systematic review	• A range of behavior change strategies noted; not enough evidence to identify which BCTs[a] linked to efficacy<br>• Multi-component interventions that combine apps with other intervention strategies appear to be more effective than stand-alone app<br>• Targeting & tailoring apps to specific population groups may enhance efficacy of app-based intervention
Quelly et al [25] (2016)	Impact of mobile apps to combat obesity in children and adolescents: A systematic literature review	• mHealth[b] apps provide motivation and assist in goal-setting behavior<br>• mHealth apps with certain features (eg, exercise instructions, encouraging messages) are more promising<br>• The type of behavior feedback in important: a study involving caring for a virtual pet which expressed both positive and negative feedback was more motivating than when provided only positive feedback

Schoeppe et al [1] (2017)	Apps to improve diet, physical activity and sedentary behavior in children and adolescents: a review of quality, features and behavior change techniques	• Frequently used BCTs[a] were providing 'instructions', 'general encouragement', 'contingent rewards', and 'feedback on performance'<br>• The most frequently used BCTs[a] do not reflect the most effective BCTs[a] in children: social support and modelling<br>• Attention to factors that improve children's engagement is an important app design consideration<br>• Recommended designing apps for specific child populations (eg, children, adolescents) incorporating most appropriate BCTs for that group
	Relevant original research	
Parnandi et al [27] (2015)	Development of a Remote Therapy Tool for Childhood Apraxia of Speech	• Overall positive response from children, parents and therapists on table use compared to paper-based therapy program<br>• 88% or respondents indicated they would use app multiple times per week<br>• Feedback on app design, users seeking: brighter colors, more interesting rewards, animated images, audio and visual prompts<br>• Ongoing are needed to maintain app novelty; researchers plan to incorporate plans and puzzles into therapy activities in future versions
Janko et al [28] (2017)	E-Gibalec: Mobile application to monitor and encourage physical activity in schoolchildren	• App provides selection of developmentally appropriate avatars to represent users that convey emotions depending on progress towards goal as an internal motivation strategy<br>• Focus group found children preferred animals to boy/girl avatars<br>• Two different reward systems for completing exercises<br>• Interaction interface for parents and physical education teachers to monitor physical activity and update goals
Patten et al [29] (2017)	A pilot study of children's physical activity levels during imagination-based mobile games	• Gamified series of apps for promoting moderate to vigorous activity at outdoor playground with parental assistance<br>• Focus in design on building self-efficacy by providing wide range of interactions with playground to maximize feeling of engagement,

		contributions to play, and experiencing success<br>• Social support is linked to higher levels of physical activity, therefore app designed to have both adult and child as active game participants<br>• App designed to encourage physical play while discouraging screen fixation, including prompts for parents to hold device and guide play
Boster and McCarthy [30] (2018)	Designing augmentative and alternative communication applications: the results of focus groups with speech-language pathologists and parents of children with autism spectrum disorder	• Animation and customization were primary appealing features in AAC[c] devices, with animation as a feedback mechanism recommended<br>• App designs with popular game characteristics and characters were seen as appealing and beneficial for children<br>• Embedded videos for instructional purposes will help children learn, especially where customized to the child's ability

[a]BCTs: behavior change techniques

[b]mHealth: mobile health

[c]AAC: Augmentative and alternative communication
